# Disruption of nuclear speckles reduces chromatin interactions in active compartments

**DOI:** 10.1186/s13072-019-0289-2

**Published:** 2019-07-17

**Authors:** Shibin Hu, Pin Lv, Zixiang Yan, Bo Wen

**Affiliations:** 1 MOE Key Laboratory of Metabolism and Molecular Medicine, School of Basic Medical Sciences, and Institutes of Biomedical Sciences, Fudan University, Shanghai, 200032 China; 2 The Fifth People’s Hospital of Shanghai, and Institutes of Biomedical Sciences, Fudan University, Shanghai, 200032 China; 3 MOE Key Laboratory of Metabolism and Molecular Medicine and Department of Biochemistry and Molecular Biology, School of Basic Medical Sciences, Fudan University, Shanghai, 200032 China

**Keywords:** Nuclear speckles, 3D genome, Nuclear architecture, SRRM2, Hi-C

## Abstract

**Background:**

Nuclei of eukaryotes contain various higher-order chromatin architectures and nuclear bodies (NBs), which are critical for proper nuclear functions. Recent studies showed that active chromatin regions are associated with nuclear speckles (NSs), a type of NBs involved in RNA processing. However, the functional roles of NSs in 3D genome organization remain unclear.

**Results:**

Using mouse hepatocytes as the model, we knocked down SRRM2, a core protein component scaffolding NSs, and performed Hi-C experiments to examine genome-wide chromatin interactions. We found that *Srrm2* depletion disrupted the NSs and changed the expression of 1282 genes. The intra-chromosomal interactions were decreased in type A (active) compartments and increased in type B (repressive) compartments. Furthermore, upon *Srrm2* knockdown, the insulation of TADs was decreased specifically in active compartments, and the most significant reduction occurred in A1 sub-compartments. Interestingly, the change of intra-TAD chromatin interactions upon Srrm2 depletion was not associated with the alteration of gene expression.

**Conclusions:**

We show that disruption of NSs by *Srrm2* knockdown causes a global decrease in chromatin interactions in active compartments, indicating critical functions of NSs in the organization of the 3D genome.

**Electronic supplementary material:**

The online version of this article (10.1186/s13072-019-0289-2) contains supplementary material, which is available to authorized users.

## Background

The nuclei of higher eukaryotes contain various 3D architectures, which are critical for proper nuclear functions such as transcription and RNA processing. As revealed by Chromosome Conformation Capture (3C)-based techniques, interphase chromatin organizes into multi-scale 3D structures including genomic compartments, topologically associating domains (TADs), and chromatin loops [1, 2]. The compartments consist of A and B types, which correspond to active and repressive chromatin, respectively. Via high-resolution Hi-C maps, these two types of compartments can be further partitioned into six sub-compartments (A1, A2, and B1–B4), which are enriched by distinct epigenetic markers [3]. TADs are self-associated chromatin domains, which are thought to be functional units of the genome [4, 5].

The molecular mechanism of loop domains formation has been revealed in recent years. CTCF and cohesin were proposed to be essential proteins for the loop domains, following chromatin extrusion model [6]. Indeed, the acute degradation of CTCF or cohesin led to the loss of TADs or loop domains [7, 8]. Interestingly, compartments were not affected by the depletion of these proteins, suggesting that the mechanisms underlying TADs and compartments are independent.

Besides the higher-order chromatin, the nucleus contains many nuclear bodies (NBs). These NBs are dynamic, membrane-less architectures, which are involved in various nuclear functions [9]. It has been shown that some NBs are responsible for the organization of the 3D chromatin architecture. For example, transcription factories (clustering of highly transcribed genes at transcriptional hotspots) can loop out of active genes from the core chromosome territory [10]. About 4% of the genomic regions are associated with the nucleolus (nucleolus-associated chromatin domains, NADs), which contain gene families and satellite repeats [11]. Highly expressed histone and U sn/snoRNA gene clusters are associated with Cajal bodies [12], which are essential for the maturation of mRNAs of histone genes.

Nuclear speckles (NSs), also known as interchromatin granule clusters (IGCs), are nuclear domains in the interchromatin regions of the nuclei, which are enriched in various snRNPs, splicing factors and other factors involved in mRNA 3′ end processing and m6A modification [13]. Recent studies further demonstrate that active chromatin regions are physically associated with NSs [14–16]. However, the functional roles of NSs in 3D chromatin organization remain unclear.

NSs contain hundreds of proteins, including many splicing factors [17]. Among them, Srrm2 (SRm300) is one of the core structural components of NSs, and knockdown of SRRM2 disrupted the speckle-type staining of SC35 and MALAT1 RNAs [18]. In this study, we disrupted NSs by depleting Srrm2 and examined the impacts on 3D chromatin organization at the genome-wide scale.

## Results

### Knockdown of *Srrm2* disrupts NSs and changes the expression of more than one thousand genes

We knocked down *Srrm2* gene expression in mouse hepatocytes (AML12 cell line) with two short-hairpin RNAs (shRNAs). *Srrm2* was knocked down by two independent shRNAs, which lead to a significant reduction in mRNA and protein levels of Srrm2 (Additional file 1: Fig. S1a, b). It was also observed that there were strong signal reductions of Srrm2 in the immunofluorescence analysis (Additional file 1: Fig. S1c). As expected, *Srrm2* knockdown abolished most of the SC35-staining foci (Additional file 1: Fig. S1c, e). We stained with an unrelated protein HNRNPU that did not change between conditions (Additional file 1: Fig. S1d). Furthermore, *SRRM2* knockdown in human osteosarcoma cells (U2OS) produced similar results (Additional file 2: Fig. S2). Consistently, the imaging of electron microscopy showed that the electron density of nuclear speckles became more dispersed upon SRRM2 knockdown (Additional file 3: Fig. S3). These results are consistent with those from previous studies [18], suggesting the general function of SRRM2 in the organization of NSs.

Through RNA-seq analysis, we identified 1282 differentially expressed genes between control and *Srrm2*-depleted cells (Additional file 4: Figure S4a; Additional file 7: Table S1). Gene ontology (GO) analysis indicated that the up-regulated genes (844) were linked to GO terms such as immune response and negative regulation of cell migration (Additional file 4: Fig. S4b); however, the down-regulated genes (438) were associated with hepatocyte-related functions such as triglyceride homeostasis and fatty acid metabolic process (Additional file 4: Fig. S4c). These datasets suggest that disruption of NSs caused substantial alteration of the gene expression. Of note, upon SRRM2 knockdown, the number of up-regulated genes was almost twice that of down-regulated genes, suggesting that the NS association is unnecessarily implicated in active transcription.

### *Srrm2* knockdown does not affect the formation of compartments and TADs

To investigate the impact of NS disruption on 3D chromatin organization, we performed in situ Hi-C experiments with control and *Srrm2*-depleted AML12 cells. More than 800 million valid contacts were obtained (Additional file 8: Table S2).

**Fig. 1:**
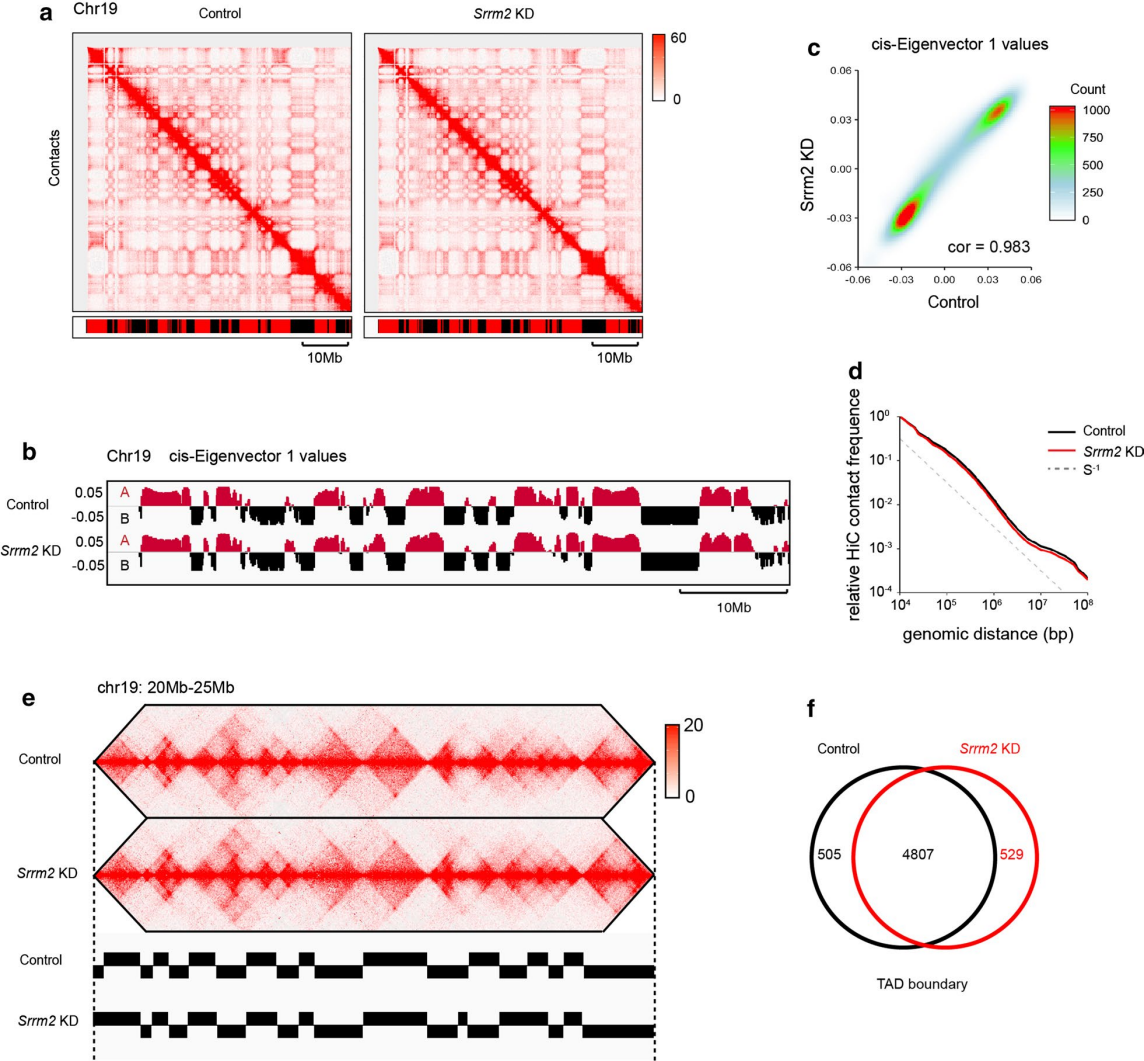
*Srrm2* knockdown does not affect the location of compartments and TADs. **a** Hi-C contact maps at 100 kb resolution across entire chromosome 19. Bar denotes segments called A (red) or B (black) compartment using 100 kb cis-eigenvector 1. Normalized Hi-C counts are multiplied by 10^6^. **b** Tracks of cis-eigenvector 1 values across the entirety of chromosome 19 are very similar between the control and *Srrm2*-depleted AML12 cells. **c** The correlation of cis-eigenvector 1 values between the control and *Srrm2*-depleted AML12 cells. Correlation coefficient (*r*) = 0.983. **d** Overall scaling of Hi-C contact frequency as a function of genomic distance is not affected by *Srrm2* depletion, showing that Srrm2 does not affect general chromatin compaction. **e** A representative region showing contacts and TAD boundaries at 10 kb resolution. **f** Venn diagrams showing the overlap of TAD boundaries between control and *Srrm2* knockdown cells

At the chromosomal scale, Hi-C contact maps appeared similar between the control and *Srrm2* knockdown samples (Fig. 1a). We then investigated the effect of *Srrm2* knockdown on genomic compartments, which are denoted by the first eigenvector (PC1) obtained through the principal component analysis of the Hi-C contact matrices. The tracks of PC1 values showed that the genomic locations of A and B compartments were largely unchanged upon *Srrm2* knockdown (Fig. 1b). Consistently, the PC1 values of control and *Srrm2*-depleted cells were highly correlated (Fig. 1c, *r* = 0.983). Furthermore, the scaling of contact frequencies as a function of genomic separation was not changed (Fig. 1d). These data indicate that the genomic compartments are largely unchanged upon *Srrm2* knockdown.

Next, we examined the effect of *Srrm2* knockdown on TADs. The insulation method (boundary score > 0.3) was used to define TAD boundaries for each replicates in control and SRRM2-depleted samples (Fig. 1e; Additional file 9: Table S3). If the distance between two boundaries was less than or equal to 2 bins (20 kb), then they were merged as one boundary. Using these criteria, we found that 89.4–90.4% boundaries overlapped between the replicates for each condition, and thus, we used these overlapped boundaries (control: 5312 boundaries; knockdown: 5335 boundaries) for further analysis. With the same criteria, we revealed that there were 90.1–90.5% TAD boundaries that overlap between the conditions (Fig. 1f). This overlap ratio was slightly higher than the ratio between replicates, indicating that the false-positive rate of boundary difference was high. It was thus concluded that TAD boundaries are largely unchanged, and TAD boundaries are shared between control and knockdown samples.

### *Srrm2* knockdown alters chromatin interactions in a compartment-specific manner

**Fig. 2:**
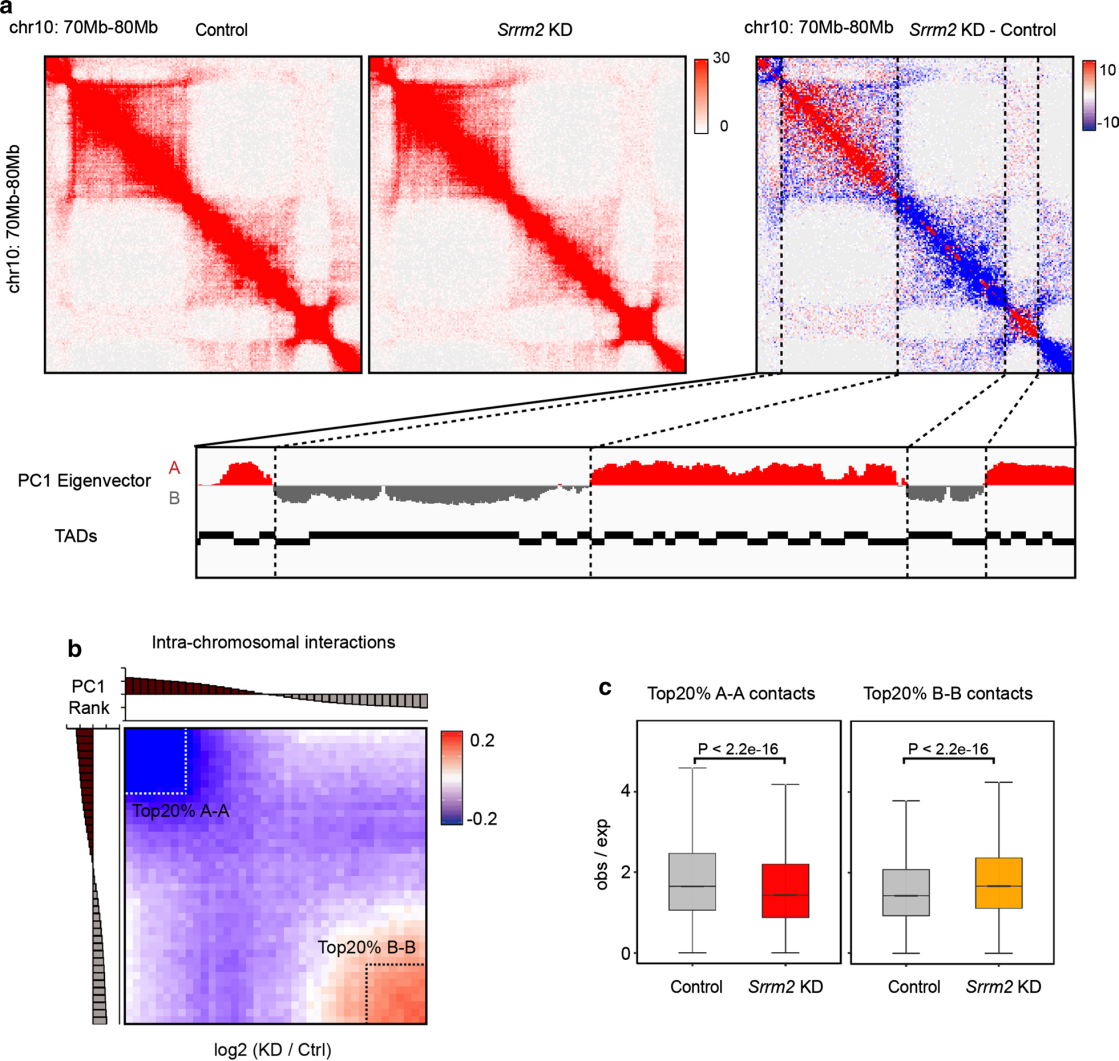
*Srrm2* knockdown alters chromatin interactions in a compartment-specific manner. **a** Snapshots of a 10 Mb region on Chr10 showing Hi-C contacts (10 kb resolution) of the control (left) and *Srrm2* knockdown (middle) AML12 cells. The differential contact map (right) showing that chromatin interactions are decreased (blue) and increased (red) in compartments A and B, respectively. Normalized Hi-C counts are multiplied by 10^6^. **b** Genomic bins (100 kb) ranked by cis-eigenvector 1 values against the intra-chromosomal interaction matrix (log2 ratios). **c** The quantification of chromatin contacts between top 20% A compartments (left) and top 20% B compartments (right) upon *Srrm2* knockdown. *P* values: Wilcoxon rank-sum test

We then studied the impact of *Srrm2* knockdown on intra-chromosomal interactions. By comparing the intra-chromosomal contact matrices and the location of compartments, we found that the trend of interaction changes differed between the two types of compartments: decreased in A and increased in B compartments (Fig. 2a). To further explore the relationship between interaction changes and compartmental intensity, we ranked the PC1 values of the control cells from high to low and averaged them into 40 intervals. Based on calculated average fold change of chromatin interactions in each interval upon *Srrm2* knockdown, results showed that the regions with the largest decrease (increase) in interaction were enriched in A (B) compartments with the highest (lowest) PC1 values (Fig. 2b). Furthermore, the chromatin interactions in the top 20% A compartments were significantly decreased, while those in the top 20% B compartments were significantly increased (Fig. 2c).

### Identification of active sub-compartments in AML12 cells

Previous studies showed that inter-chromosomal interactions and highly active chromatin regions (sub-compartment A1) are associated with NSs [13, 14]. To better understand the effect of *Srrm2* knockdown on the 3D chromatin organization, we characterized A1 and A2 sub-compartments according to the inter-chromosomal contacts in AML12 cells (Additional file 10: Table S4).

The contact domain structure was regarded as the basic unit of the compartment. We used two steps to assign the contact domains to sub-compartments A1, A2 and B. Firstly, the contact domains were assigned to either A or B compartment using the PC1 eigenvectors at 10 kb resolution. The average PC1 values for each contact domain were then calculated; if the domain’s average PC1 score was greater than zero, it was defined as A-domain. Otherwise (if less than zero), the domain was defined as B-domain.

Secondly, the inter-chromosomal contact information was used to divide A-domain into A1-domain and A2-domain further. Then, the average inter-chromosomal interaction was calculated (O/E) between A-domain and 20 new contact matrix for each chromosome were generated (e.g., chr1-chrX). In the new matrix, each row represents the A-domain in one chromosome (e.g., chr1 A-domain 1-200), each column represents the A-domain in the other chromosomes (e.g., chr2-chrX A-domain), and each data point in the matrix represents the average interaction (O/E) between A-domains in different chromosomes (e.g., chr1 No. 100 A-domain vs chr2 No. 200 A-domain). Next, the rows of these matrices were clustered by *K*-means clustering (*K* = 2); this procedure divided A-domain into two clusters. We then calculated the enrichment of chromatin marks (H3K27ac; H3K9ac) in the two clusters for each chromosome. Finally, we defined the more active clusters as A1 and less active clusters as A2 for each chromosome. Each A1 cluster in A-domain was defined as A1-domain; otherwise, they were designated as A2-domain.

**Fig. 3:**
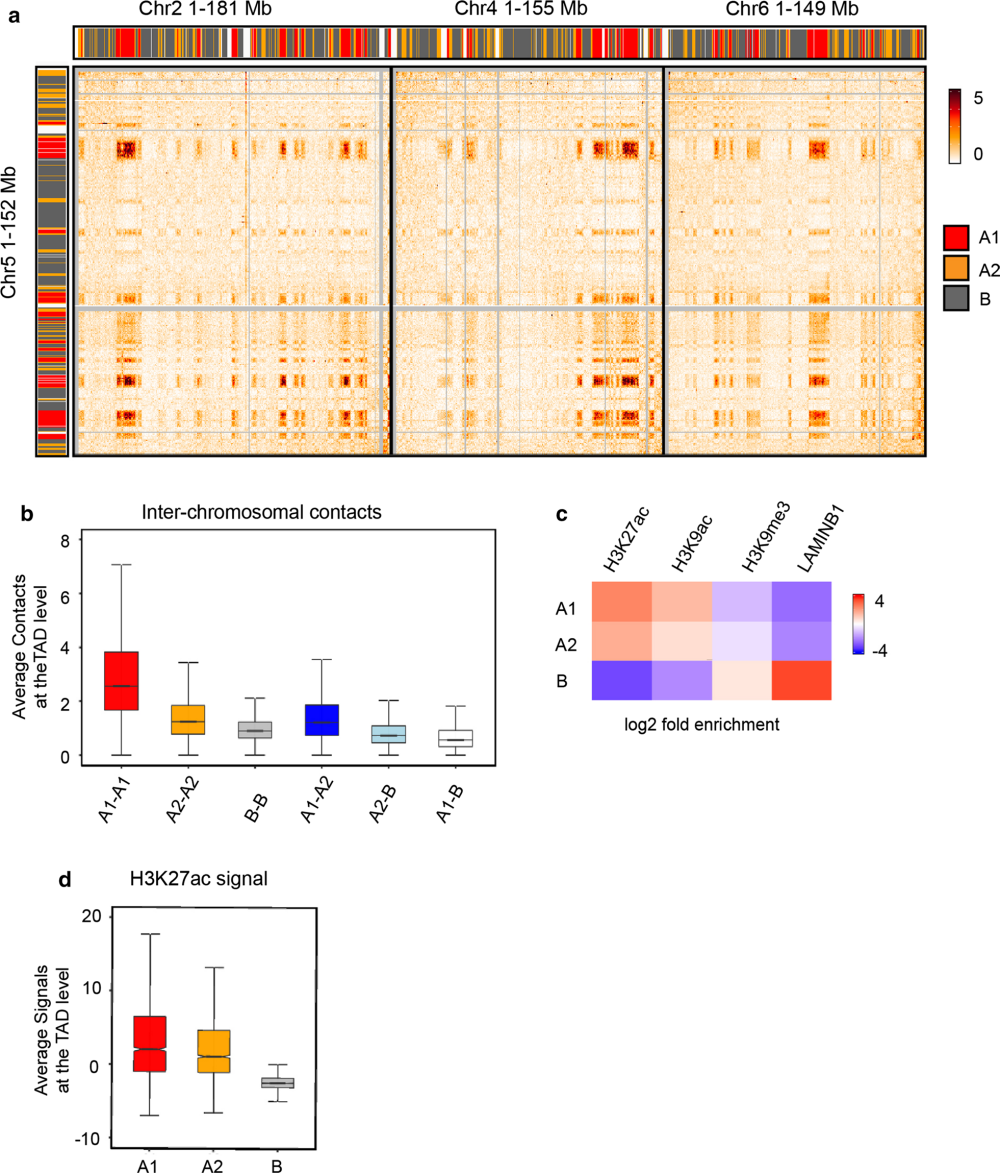
Identification of sub-compartments A1 and A2 in AML12 cells. **a** A heat map showing inter-chromosomal contacts between Chr5 and other three chromosomes (Chr2, Chr4 and Chr6). Color bars denote A1 sub-compartments (red), A2 sub-compartments (orange) and B compartments (gray). **b** Quantification of inter-chromosomal contacts within and between (sub)-compartments at the TAD level. **c** Heat map showing enrichment of the histone modifications (H3K27ac, H3K9ac and H3K9me3) and Lamin B1 in (sub)-compartments. **d** Quantification of H3K27ac signals at the TAD level

The inter-chromosomal contacts were most apparent among A1 sub-compartments (Fig. 3a). Quantitatively, A1 sub-compartments displayed the highest degree of inter-chromosomal interactions at the TAD level (Fig. 3b). Furthermore, A1 sub-compartments contained the highest levels of active chromatin markers such as H3K27ac (Fig. 3c, d), consistent with the previously reported results [3].

### *Srrm2* knockdown specifically decreases the insulation of TADs in active compartments

**Fig. 4:**
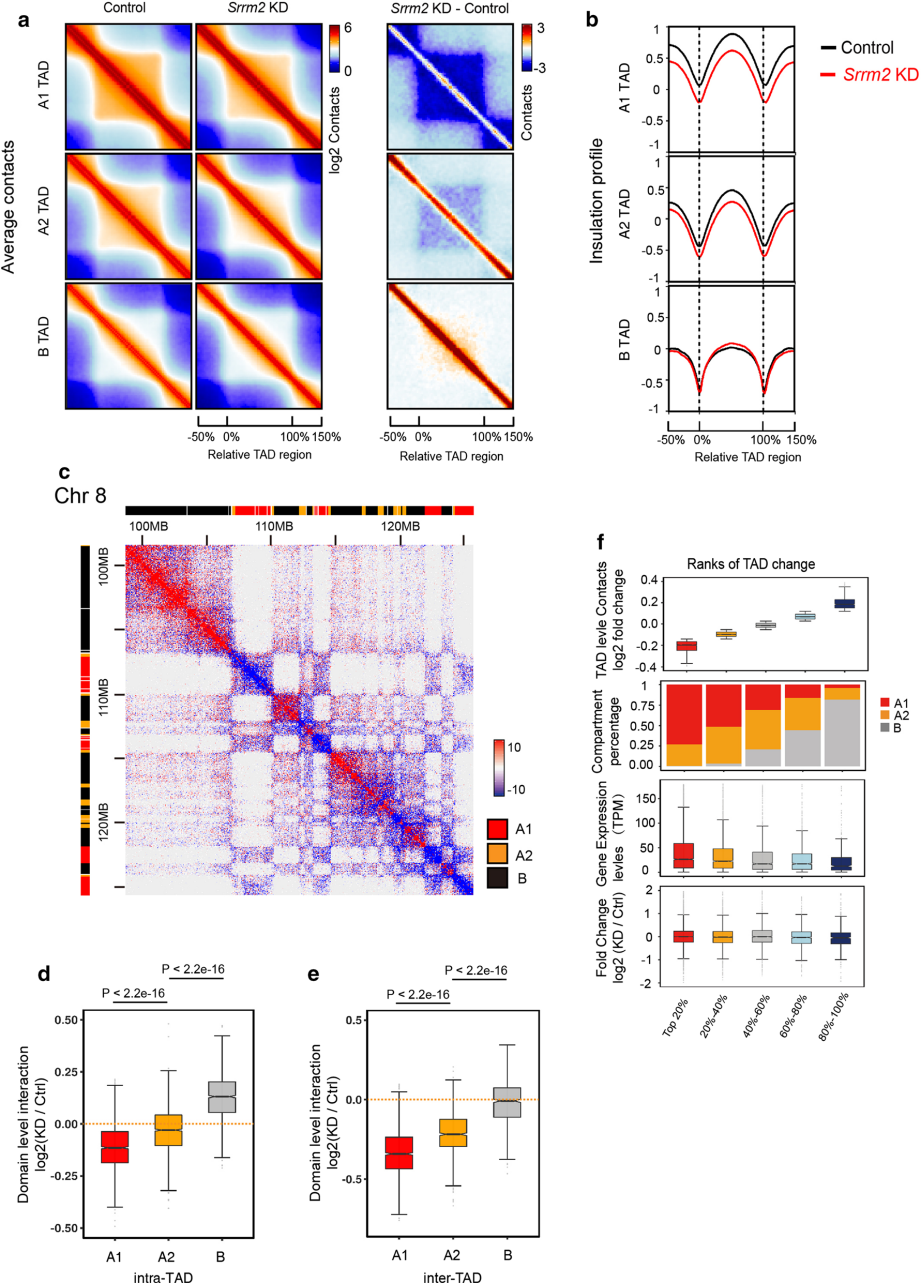
*Srrm2* knockdown specifically decreases the insulation of TADs in active compartments. **a** Average contact maps of control (left) and Srrm2 knockdown (middle) cells, and differential contact maps of TADs in A1, A2 sub-compartments, and B compartments. **b** Average insulation profiles of TADs in the three (sub)-compartments. **c** Snapshots of a 27 Mb region on Chr8 showing that intra-chromosomal TAD interactions are decreased (blue) and increased (red) in compartments A1, A2 and B, respectively. Normalized Hi-C counts are multiplied by 106. **d**, **e** Quantification of the intra-TAD (**d**) and inter-TAD (**e**) interactions in different categories. *P* values: Wilcoxon rank-sum test. **f** The relationship between the intra-TAD fold change, (sub)-compartments, RNA expression levels, and the changes of RNA expression upon *Srrm2* knockdown

We then divided TADs into three categories in the context of (sub)-compartments: A1, A2 and B. By calculating the average interaction intensity of these three groups of TADs, we found that *Srrm2* knockdown caused a substantial reduction in the interaction within the A1 sub-compartments (Fig. 4a). We further calculated the average insulation curve for these TADs and the surrounding regions. The insulation curves of A1-TADs and A2-TADs decreased both inside and outside the TADs, while those of B-TADs were not significantly changed (Fig. 4b). We have provided three examples, which clearly show that chromatin interactions were decreased in A1 sub-compartments focally (Fig. 4c; Additional file 6: Fig. S6). This indicates that the *Srrm2* knockdown weakened both the intra- and inter-TAD interactions within A1 and A2 sub-compartments. We further compared the degree of interaction changes of these three groups of TADs: the decrease in intra- and inter-TAD interactions was the most significant in sub-compartment A1 upon *Srrm2* knockdown (Fig. 4d, e).

Next, the relationship between intra-TAD interactions and gene expression was investigated (Fig. 4f). The TADs (five groups in total) were ranked according to the magnitude of the intra-TAD decreases upon *Srrm2* knockdown: the higher the ranking, the greater the decrease. With the decrease in the rank, the percentages of A1 and A2 sub-compartments declined, while the percentages of B compartments increased. Consistently, the gene expression levels in control cells reduced with the ranking. However, the relative gene expression upon Srrm2 knockdown remained unchanged in all groups, suggesting that alterations in intra-TAD chromatin interactions did not change gene expression.

## Discussion

**Fig. 5:**
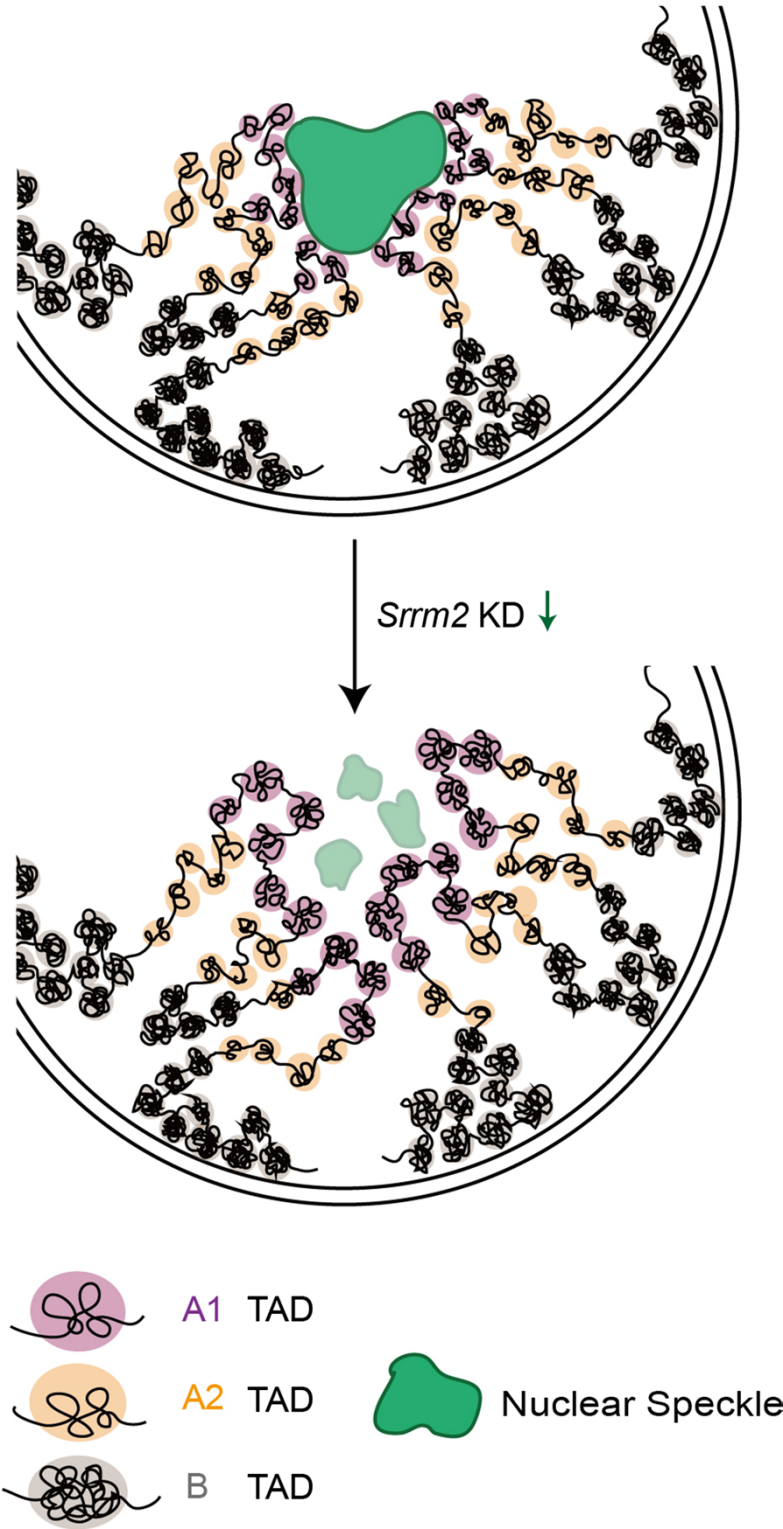
A model on how NSs maintain 3D genome organization. Highly active A1 sub-compartments are distributed around NSs. Upon the disruption of NSs, interactions within A1 regions are decreased substantially, and the 3D genome organization of other compartments is further remodeled

Recent investigations revealed that highly active chromatin regions are physically associated with NSs [14–16] and that repressive chromatin regions associate with the nuclear periphery or the nucleolus [11, 19]. In this study, we demonstrate that disruption of NSs by *Srrm2* knockdown reduces chromatin interactions at active chromatin regions, and the most substantial decreases occur in the A1 sub-compartments. Based on these findings, we propose a model on how NSs participate in the 3D organization of chromatin (Fig. 5). The active A1 sub-compartments are connected to the NSs, while the repressive chromatin regions are anchored to the nuclear membrane or the nucleolus, thereby restraining the random movement of the chromosomes in the nucleus. When the NSs are disrupted, the A1 sub-compartments are partially disorganized, leading to the decrease in intra- and inter-TAD interactions in A1 sub-compartments, and subsequently changing chromatin interactions in other compartments.

We found that *Srrm2* knockdown changes the expression of 1282 genes, of which 438 were significantly down-regulated and 844 were significantly up-regulated. However, these changes in gene expression are not associated with changes in chromatin interactions at the level of TAD. These results may imply that: (1) although TAD interactions were reduced in A1 sub-compartments, the functions of TADs as transcription units were preserved; (2) changes in gene expression may be due to secondary effects resulting from changes of chromatin organization.

Although the genomic regions close to NSs are associated with higher levels of Pol II binding, it remains unclear whether this association is implicated in gene activation. In this study, we showed that disruption of NSs by SRRM2 knockdown leads to the decrease in intra-TAD interactions at A1 sub-compartments. However, no significant gene expression changes were found to be associated with these chromatin changes. These results suggested that there was no direct cause-and-effect relationship between NS association and the transcriptional activity of individual genes.

Interestingly, targeted degradation of cohesin removed most of the chromatin loops, but only a small number of genes in the super-enhancers were changed [7]. Furthermore, only 370 genes were affected by acute CTCF degradation [8]. Therefore, the relationship between gene expression regulation and spatial organization of the genome remains to be further studied. The recently established CRISPR-GO system [20] may be a useful tool to answer this question.

As one of the major types of nuclear bodies in the nucleus, the NS is associated with various proteins and RNAs [13]. Through mass spectrometry, 200–300 NS-associated proteins were identified, including snRNPs, SR proteins and kinases [21]. Besides, MALAT1 and U1 RNAs are also localized in NSs [22]. Based on the super-resolution microscopy, MALAT1 and U1 were localized at the periphery of the NSs, while SC35 and SON are localized at the core [23]. Upon *Srrm2* knockdown, the localization of MALAT1, U1 snRNAs, poly(A)^+^ RNAs and SC35 became scattered, indicating that Srrm2 is involved in organizing NSs [18], and this was confirmed in this study. Srrm2 contains a broad disordered region (VSL2 predictor from PONDR: 100% disordered of 2703 amino acid residues), suggesting the propensity to initiate the phase separation. It would be interesting to test whether Srrm2 organizes the NSs by liquid–liquid phase separation. Nevertheless, as demonstrated in this study, disruption of NSs by Srrm2 knockdown facilitated exploration of the functional roles of NSs in the nuclear architecture and 3D genome organization.

Besides, given that NSs contain many components, including proteins and noncoding RNAs, disrupting NSs by depleting other molecules may change other aspects of the 3D genome organization. Further efforts would be needed to fully decipher the roles of NSs in nuclear architecting and their physiological and pathological relevance.

## Conclusions

We demonstrate that disruption of NSs by *Srrm2* knockdown reduces chromatin interactions in active chromatin compartments, especially in the A1 sub-compartments. These results indicate essential functions of NSs in the organization of the 3D genome.

## Materials and methods

### Cell lines and culture

The mouse hepatocyte cell line alpha mouse liver 12 (AML12) and human osteogenic sarcoma cell (U2OS) were obtained from ATCC. AML12 cells were cultured in DMEM/F12 (HyClone) supplemented with 10% fetal bovine serum (FBS, Biowest), ITS Liquid Media Supplement (Sigma) and 0.1 μM dexamethasone (Sigma) at 37 °C and 5% CO_2_. U2OS cells were cultured in DMEM (Dulbecco’s modified Eagle’s medium, HyClone) supplemented with 10% fetal bovine serum (FBS, Biowest), nonessential amino acids (Gibco) at 37 °C and 5% CO_2_. AML12 and U2OS cells were authenticated by STR profiling.

### Knockdown experiments

For short-hairpin RNA (shRNA)-based knockdown, AML12 or U2OS cells were infected with shRNA-containing lentivirus as previously described [21]. Target sequences of shRNAs are as follows: mouse Srrm2-1, CCCAAACCATACAGCCTTGTT; mouse Srrm2-2, CCAGTTTATCTCCAGAACATA; human SRRM2-1, CCCAAAGTGAAGGCAATAATA; human SRRM2-2, CGCCACCTAAACAGAAATCTA.

### Immunofluorescence assay

Cells were fixed in 4% formaldehyde for 10 min at room temperature prior to cell permeabilization with 0.5% Triton X-100 (RT; 10 min). The cells were blocked with phosphate-buffered saline (PBS) containing 4% bovine serum albumin (BSA) for 0.5 h at room temperature and processed for immunostaining. Staining was done using mouse anti-SC35 (1:500, Abcam, ab11826), rabbit anti-SRRM2 (1:200, Novus Biologicals, NBP2-55697), rabbit anti-HNRNPU (1:200, Abcam, ab20666) and donkey anti-mouse Alexa Fluor 488 (1:600, Jackson ImmunoResearch Laboratories). Fluorescence images were taken and analyzed with a Leica confocal microscope (TCS SP5; Leica, Germany).

### Quantitative RT-PCR (qRT-PCR)

RNA extraction and qRT-PCR were conducted as described [24]. The mRNA expression levels for the genes of interest were compared with that of β-actin. Sequences of primers are as follows:

- Mouse_Srrm2_F:CTGCAAGAATGTCCCAGGTT;
- Mouse_Srrm2_F:ATGCCGGAATAGCAGATGTC;
- Human_SRRM2_F:CTGACTCTGCTTCCTCCTCC;
- Human_SRRM2_F:CTGAAAGGCGCATCTCCCT;
- Mouse_Actb-F:GGTCATCACTATTGGCAACG;
- Mouse_Actb-R:ACGGATGTCAACGTCACACT;
- Human_ATCB-F:ACTCTTCCAGCCTTCCTTCC;
- Human_ATCB-R:TGTTGGCGTACAGGTCTTTG.

### Western blotting

The cell lysates were blotted against primary antibodies as follows: anti-β-actin (1:10,000, AOGMA, AGM11086) and rabbit anti-SRRM2 (1:1000, Novus Biologicals, NBP2-55697). The blots were visualized with peroxidase-coupled secondary antibodies.

### Electron microscopy analysis

Electron microscopy experiments were performed as described [25, 26]. AML12 cells were scratched and collected after fixing by 2.5% glutaraldehyde in phosphate-buffered saline (PBS; Corning, R21-040-CV). Cells were centrifuged to the bottom of the tube and then embedded with agar. The agar was cut into 1-mm^3^ size samples for further treatment with 1% osmic acid in PBS and gradient dehydration with alcohol. The sample was treated with a mixture of epoxypropane (Sinopharm Chemical Reagent, 80059118) and resin (Electron Microscopy Sciences, 14900), followed by embedding with pure resin. Then the sample was sliced with a diamond tool kit. Target cells that were randomly selected were captured with a transmission electron microscope (Nippon Tekno, JEOL-1230, Japan).

### In situ Hi-C and data analysis

The in situ Hi-C libraries were prepared as previously described [3]. Two biological replicates were performed for both control and Srrm2-depleted AML12 cells. The libraries were then sequenced via the Illumina HiSeq X Ten system. We used the Hi-C-pro pipeline [27] to process the Hi-C raw data. To eliminate the bias from the different sequencing depths, we randomly sampled the contacts of each sample to 200 M and then converted the sampled contacts to contact matrix at different resolutions by Juicer Tools [28]. These contact matrices were used for all downstream analysis. Next, we used the Juicer Tools to generate the PC1 eigenvectors using 100 kb resolution matrices using the following options: (java-jar juicer_tools.jar eigenvector NONE Sample1.hic 1 BP 100000). To identify contact domain boundaries, we detected boundaries and their boundary strength via the insulation method with 10 kb resolution contact matrix [29]. Then we called contact domain region according to the contact boundary location.

To assign contact domains to sub-compartments A1, A2 and B, we use the following methods. Contact domains were assigned to either A or B compartment by the PC1 eigenvectors. We calculated the average PC1 values for each contact domain; greater than zero is defined as A-domain, and less than zero is defined as B-domain. Next, we clustered A-domains by *K*-means clustering (*K* = 2) with the observed/expected ratios of inter-A-domain contacts *in trans*. Domains in each cluster exhibit distinct signatures based on the enrichment of chromatin marks. We defined more active clusters as A1 and less active clusters as A2.

### RNA-seq analysis

For RNA-seq experiments, polyA RNA-enriched and strand-specific libraries were constructed with the VAHTS Total RNA-seq (H/M/R) Library Prep Kit (Vazyme Biotech Co., Ltd), and the libraries were sequenced using the Illumina HiSeq X Ten system (Annoroad Gene Technology Corporation). We trimmed and mapped reads to the mouse mm9 reference assembly by the TopHat2 software [30] using default parameters except that we reduced maximum insertion and deletion length to 2 bp, and kept only uniquely mapped, “no mixed” and “no discordant” reads. For differential gene expression analysis, we analyzed raw read counts for GENCODE M1 genes using HTSeq [31] and then calculated statistics of differential expression via DESeq2 version 1.8.2 [32] with default parameters. To define differentially expressed genes, we used false discovery rate (FDR) 0.05 and log2 (fold change) > 1 or < − 1 as thresholds. We performed GO analysis using DAVID bioinformatics tools [33].

### Statistical analysis

All statistical calculations are included in the relevant figure legends. *P* value less than 0.05 was accepted as significant; *P < 0.05, **P < 0.01, ***P < 0.001.

## Additional files

**Additional file 1: Figure S1.** Disruption of nuclear speckles by *Srrm2* knockdown in AML12 cells. **a** Gene expression of *Srrm2* genes analyzed by RT-qPCR in AML12 cells. Error bars: s.d. of three biological replicates. ***P < 0.001, n.s. = not significant; Student’s *t* test. **b** Western blot analysis with antibodies against specified proteins; β-Actin as loading controls. **c**, **d** Immunofluorescence analyses of SRRM2, SC35 (**c**), and HNRNPU (**d**) in AML12 cells. The depletion of *Srrm2* disturbed nuclear speckles (NSs) as shown by the immunofluorescence data of the NS marker SC35. Scale bar, 10 µm. **e** Quantification of SC35 signal between control and *Srrm2*-depleted samples (Control: *n* = 118; *Srrm2* KD: *n* = 182. *P* values: Wilcoxon rank sum test). **f** Gene expression of hepatic genes analyzed by RT-qPCR in AML12 cells. Error bars: s.d. of three biological replicates. ***P < 0.001, n.s. = not significant; Student’s *t* test.

**Additional file 2: Figure S2.** Disruption of nuclear speckles by *SRRM2* knockdown in U2OS cells. **a** Gene expression of *SRRM2* genes analyzed by RT-qPCR in U2OS cells. Error bars: s.d. of three biological replicates. ***P < 0.001, n.s. = not significant; Student’s *t* test. **b** Western blot analysis with antibodies against specified proteins; β-Actin as loading controls. **c**, **d** Immunofluorescence analyses of SRRM2, SC35 (**c**), and HNRNPU (**d**) in U2OS cells. The depletion of *SRRM2* disturbed nuclear speckles (NSs) as shown by the immunofluorescence data of the NS marker SC35. Scale bar, 10 µm.

**Additional file 3: Figure S3.** Nuclear speckles were abolished after Srrm2 knockdown by electron microscopy (EM). **a** EM analysis of nuclear speckles stained by APEX fused to SC35 in AML12 cells. The image on the left was the control, on the right was Srrm2-depleted cells. Arrows point to nuclear speckles. Scale bar, 1 µm. **b** EM images of U2OS cells stained by HRP. Arrows point to nuclear speckles. Scale bar, 2 µm. **c** EM analysis of nuclear speckles in AML12 cells. The image on the left was the control, on the right was the Srrm2-depleted cell. Arrows point to nuclear speckles. Scale bar, 2 µm.

**Additional file 4: Figure S4.** Gene expression changes upon *Srrm2* knockdown. **a** Scatter plot showing gene expression changes detected by RNA-seq in *Srrm2*-depleted AML12 cells. Compared with the control, 844 genes were up-regulated, and 438 genes were down-regulated significantly. **b**, **c** Gene ontology (GO) chart of up-regulated (**b**) and down-regulated (**c**) genes. The enriched terms are ranked by − log10 (*P* value). **d** Venn diagrams show the overlap of genes between differentially spliced genes (DSGs) and differentially expressed genes. **e** Gene ontology (GO) terms of differentially spliced genes. The enriched terms are ranked by − log10 (*P* value).

**Additional file 5: Figure S5.** Calling the TAD boundaries. **a** Distance to the nearest boundary between replicates. **b** Venn diagrams show the overlap of TAD boundaries between replicates for control or *Srrm2* knockdown cells.

**Additional file 6: Figure S6.** Differential contact map of intra-chromosomal inter-TADs showing fewer (compartments A, blue) and more (compartments B, red) Hi-C signal after *Srrm2* depletion AML12 cells.

**Additional file 7: Table S1.** Differentially expressed genes upon *Srrm2* knockdown in AML12 cells.

**Additional file 8: Table S2.** Mapping statistics of the Hi-C data.

**Additional file 9: Table S3.** Coordinates (mm9) of TAD boundaries in Control and *Srrm2* knockdown samples.

**Additional file 10: Table S4.** Coordinates of sub-compartment regions in AML12 cells.

## Data Availability

The raw and processed RNA-seq and Hi-C datasets from this study are submitted to the NCBI Gene Expression Omnibus (GEO; http://www.ncbi.nlm.nih.gov/geo/) under accession numbers GSE130805 (for RNA-seq) and GSE131466 (for Hi-C).
