# Supplementary figures and images for: Disruption of nuclear speckles reduces chromatin interactions in active compartments
Source: Epigenetics Chromatin. 2019 Jul 17;12:43. doi: 10.1186/s13072-019-0289-2 (PMC6636040; doi:10.1186/s13072-019-0289-2)

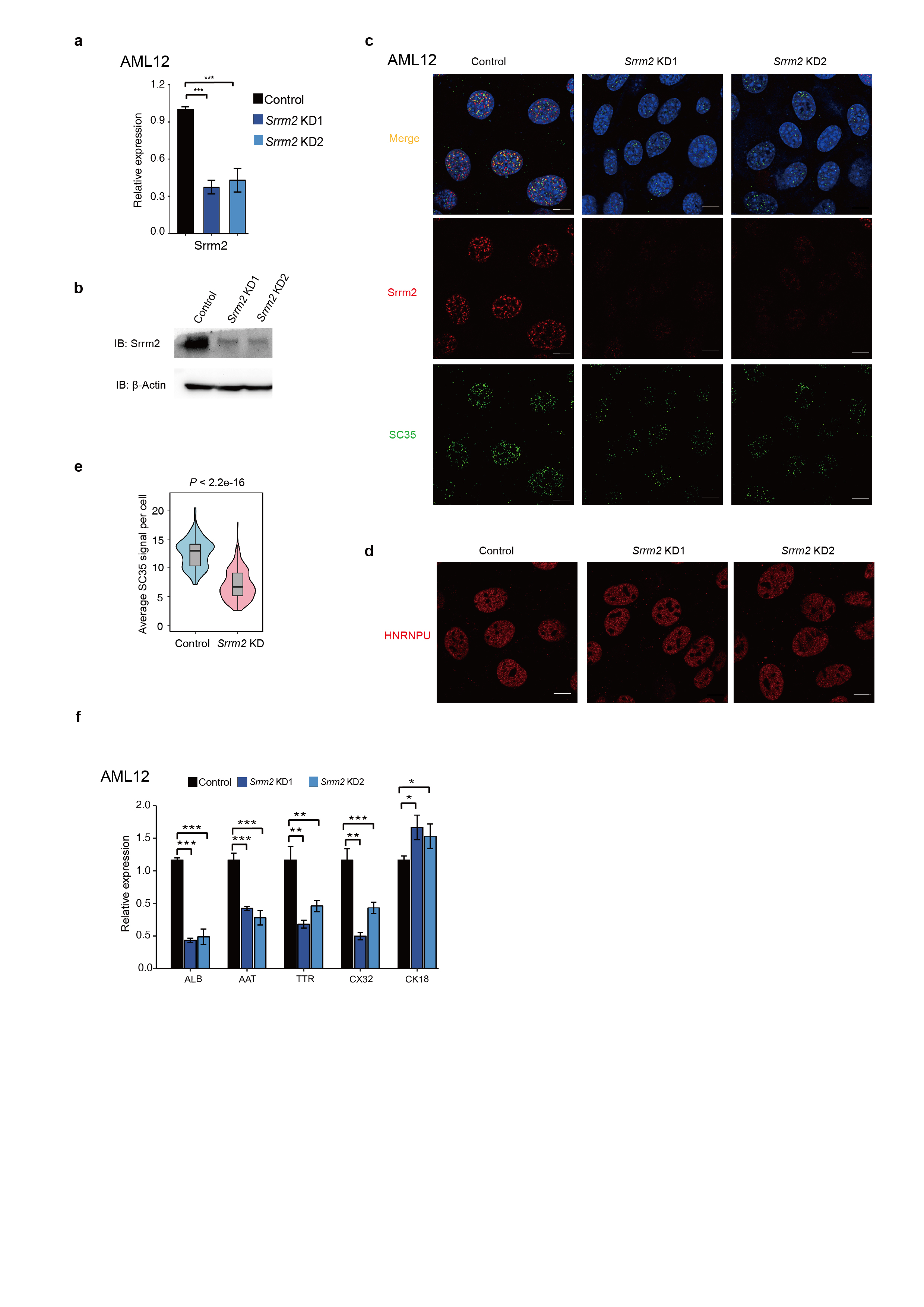

Supplement: Supplementary file 1 — Additional file 1: Figure S1. Disruption of nuclear speckles by Srrm2 knockdown in AML12 cells. a Gene expression of Srrm2 genes analyzed by RT-qPCR in AML12 cells. Error bars: s.d. of three biological replicates. ***P < 0.001, n.s. = not significant; Student’s t test. b Western blot analysis with antibodies against specified proteins; β-Actin as loading controls. c, d Immunofluorescence analyses of SRRM2, SC35 (c), and HNRNPU (d) in AML12 cells. The depletion of Srrm2 disturbed nuclear speckles (NSs) as shown by the immunofluorescence data of the NS marker SC35. Scale bar, 10 µm. e Quantification of SC35 signal between control and Srrm2-depleted samples (Control: n = 118; Srrm2 KD: n = 182. P values: Wilcoxon rank sum test). f Gene expression of hepatic genes analyzed by RT-qPCR in AML12 cells. Error bars: s.d. of three biological replicates. ***P < 0.001, n.s. = not significant; Student’s t test. [file 13072_2019_289_MOESM1_ESM.jpg]

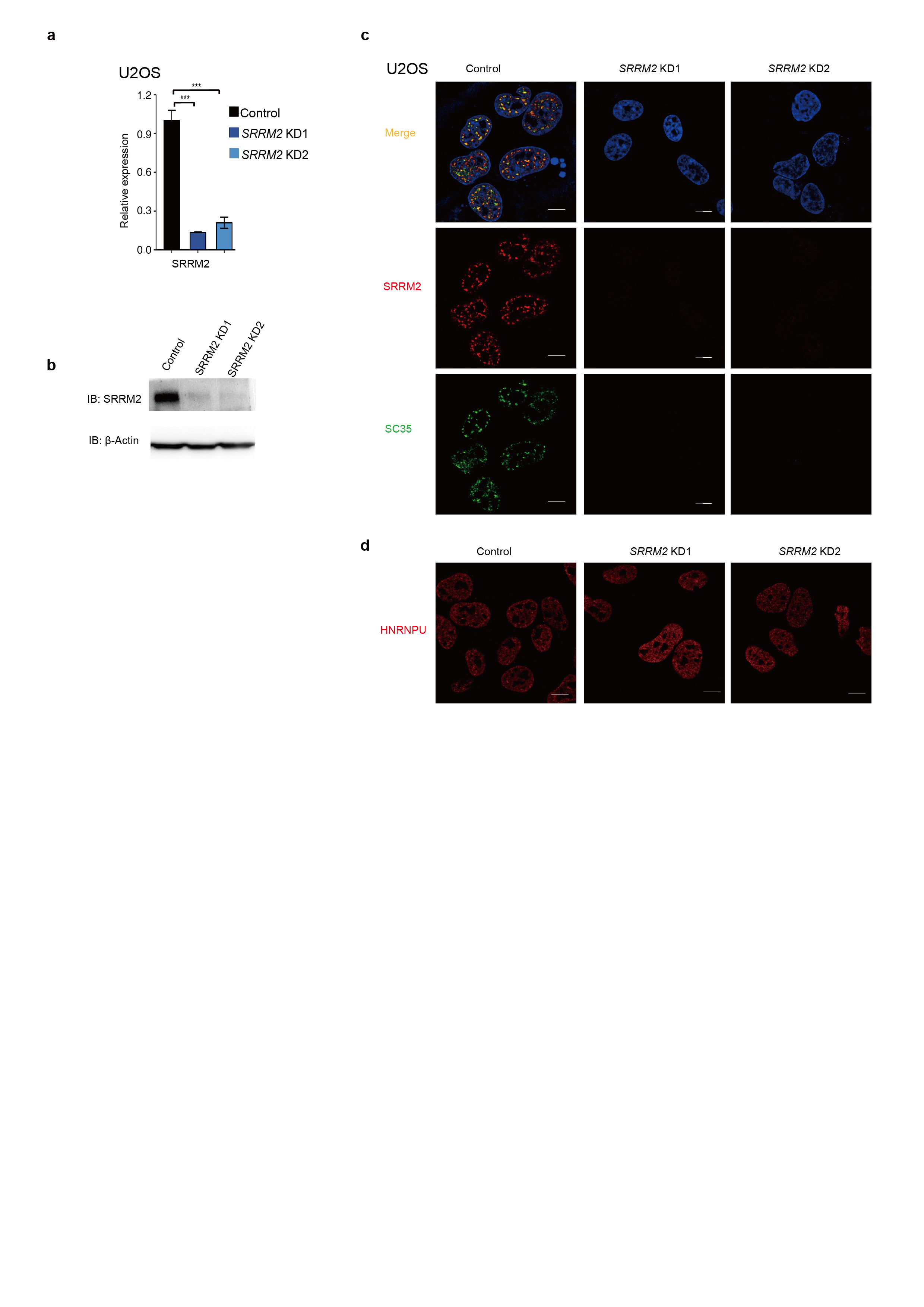

Supplement: Supplementary file 2 — Additional file 2: Figure S2. Disruption of nuclear speckles by SRRM2 knockdown in U2OS cells. a Gene expression of SRRM2 genes analyzed by RT-qPCR in U2OS cells. Error bars: s.d. of three biological replicates. ***P < 0.001, n.s. = not significant; Student’s t test. b Western blot analysis with antibodies against specified proteins; β-Actin as loading controls. c, d Immunofluorescence analyses of SRRM2, SC35 (c), and HNRNPU (d) in U2OS cells. The depletion of SRRM2 disturbed nuclear speckles (NSs) as shown by the immunofluorescence data of the NS marker SC35. Scale bar, 10 µm. [file 13072_2019_289_MOESM2_ESM.jpg]

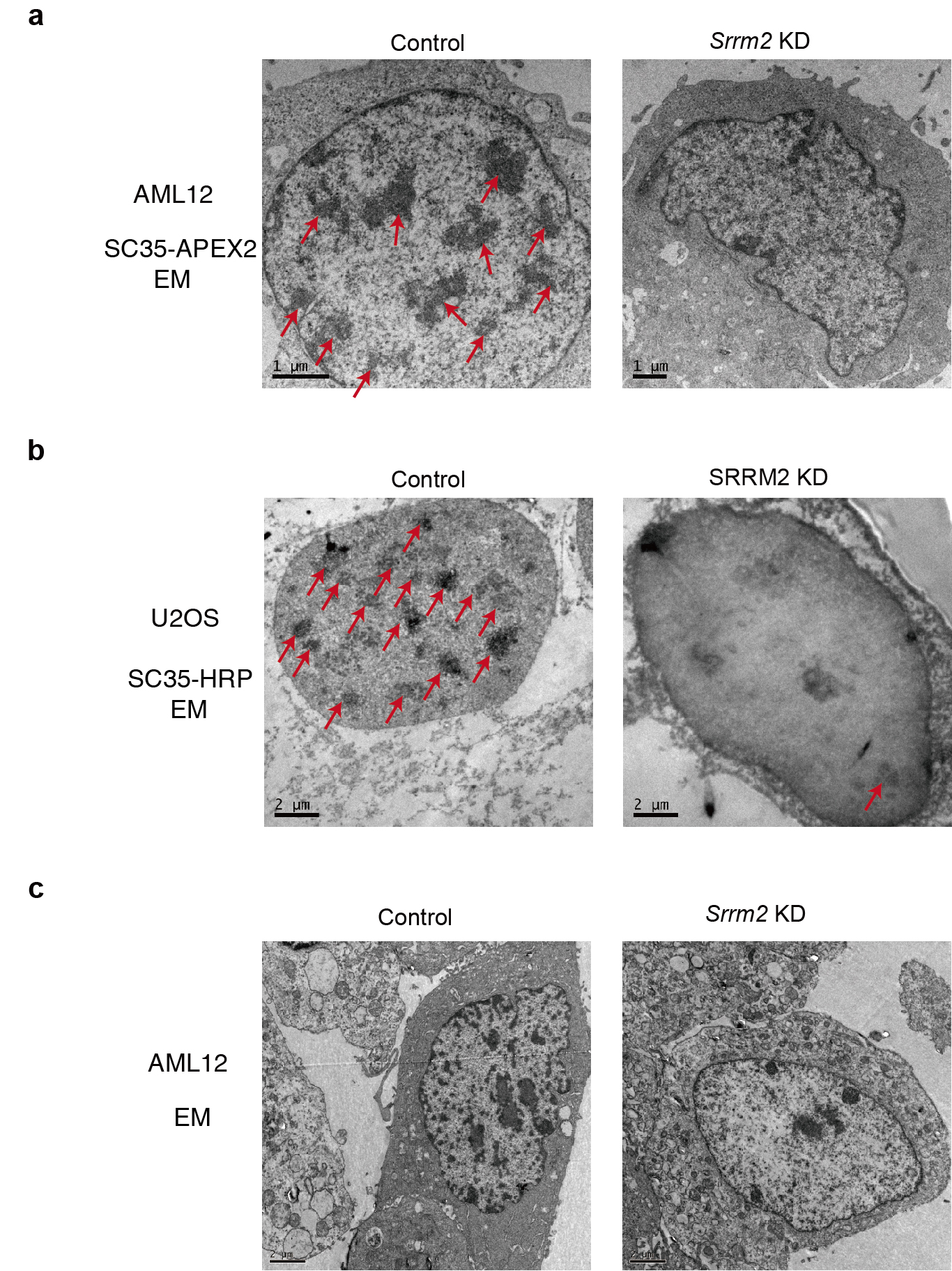

Supplement: Supplementary file 3 — Additional file 3: Figure S3. Nuclear speckles were abolished after Srrm2 knockdown by electron microscopy (EM). a EM analysis of nuclear speckles stained by APEX fused to SC35 in AML12 cells. The image on the left was the control, on the right was Srrm2-depleted cells. Arrows point to nuclear speckles. Scale bar, 1 µm. b EM images of U2OS cells stained by HRP. Arrows point to nuclear speckles. Scale bar, 2 µm. c EM analysis of nuclear speckles in AML12 cells. The image on the left was the control, on the right was the Srrm2-depleted cell. Arrows point to nuclear speckles. Scale bar, 2 µm. [file 13072_2019_289_MOESM3_ESM.jpg]

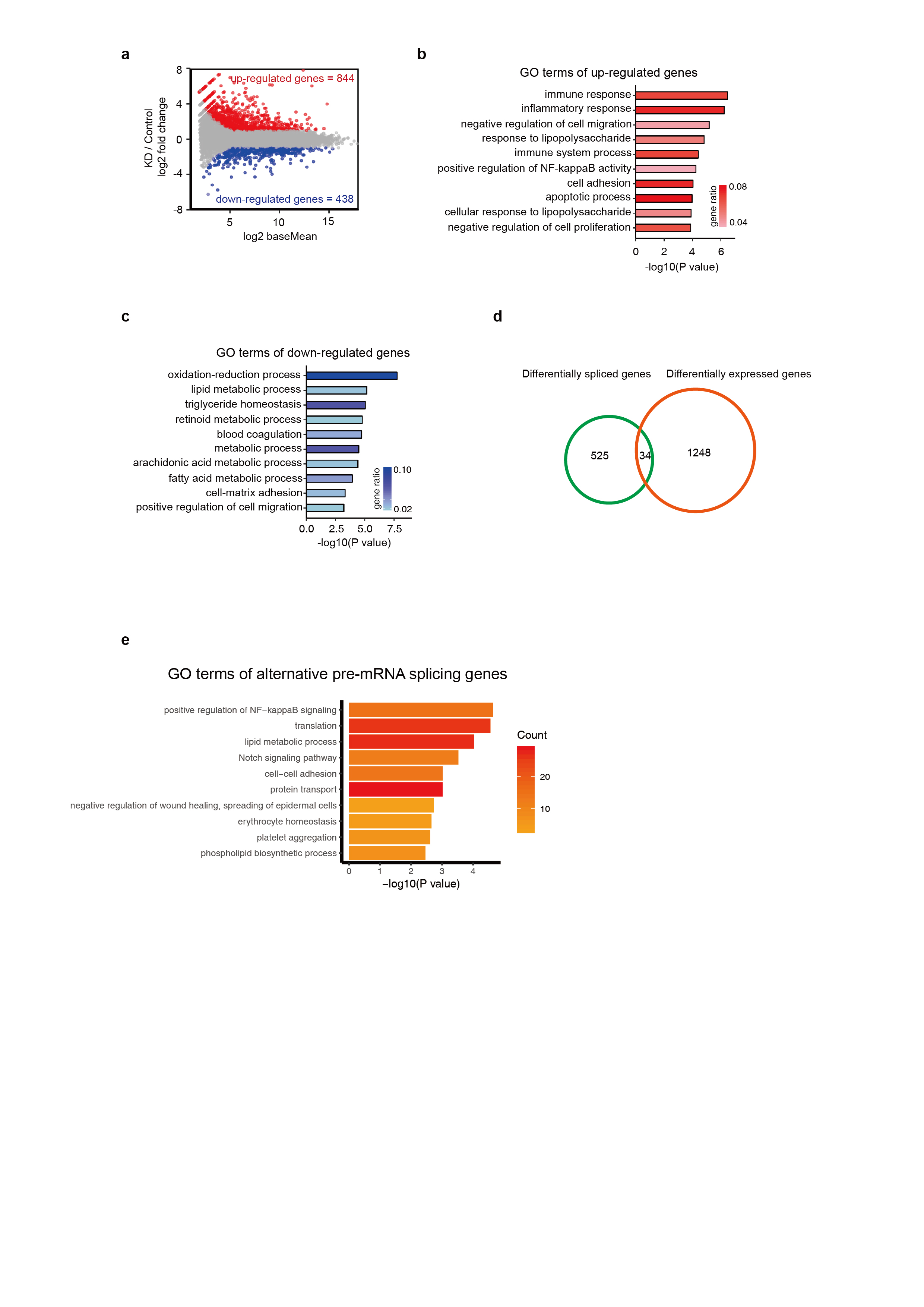

Supplement: Supplementary file 4 — Additional file 4: Figure S4. Gene expression changes upon Srrm2 knockdown. a Scatter plot showing gene expression changes detected by RNA-seq in Srrm2-depleted AML12 cells. Compared with the control, 844 genes were up-regulated, and 438 genes were down-regulated significantly. b, c Gene ontology (GO) chart of up-regulated (b) and down-regulated (c) genes. The enriched terms are ranked by − log10 (P value). d Venn diagrams show the overlap of genes between differentially spliced genes (DSGs) and differentially expressed genes. e Gene ontology (GO) terms of differentially spliced genes. The enriched terms are ranked by − log10 (P value). [file 13072_2019_289_MOESM4_ESM.jpg]

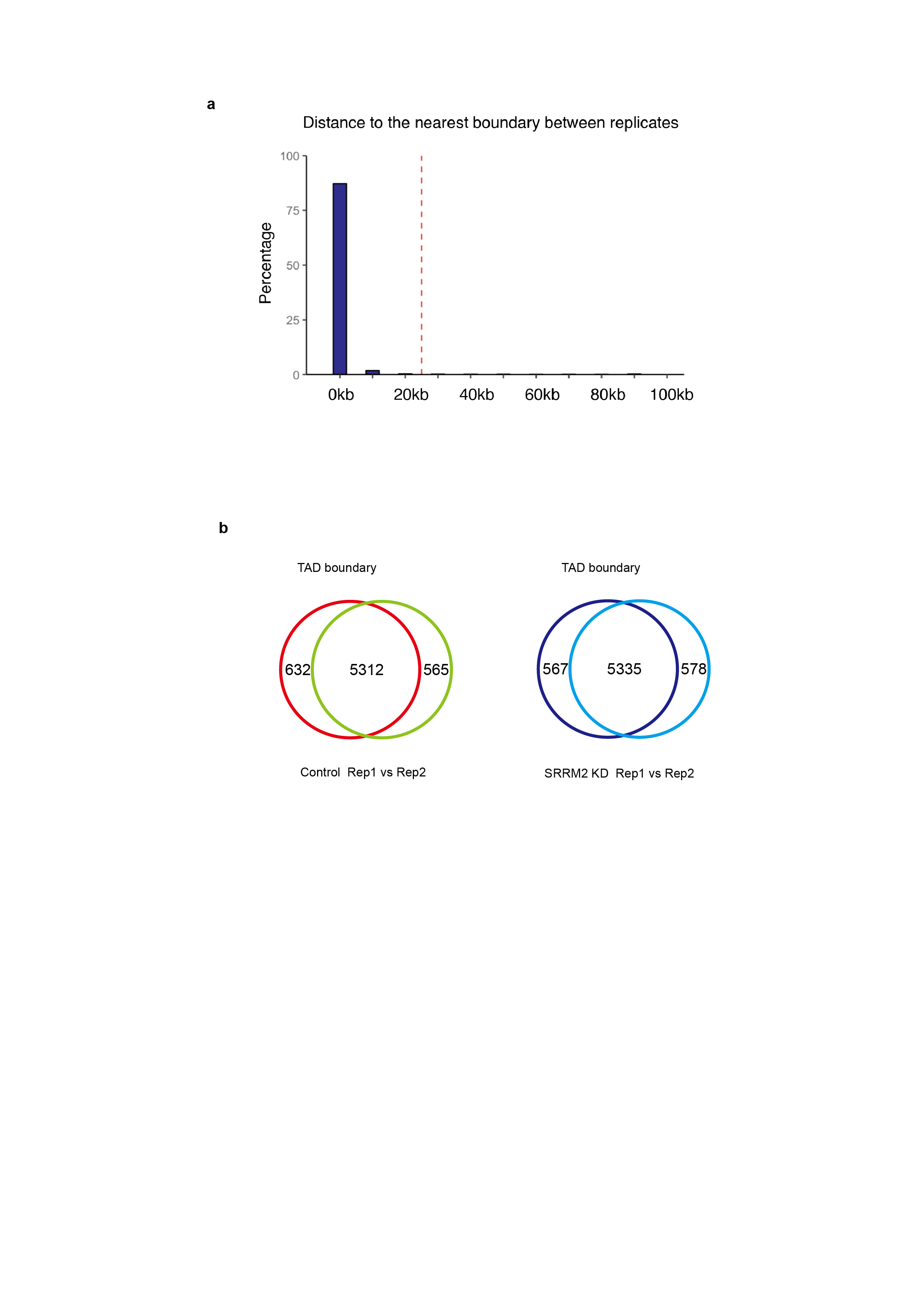

Supplement: Supplementary file 5 — Additional file 5: Figure S5. Calling the TAD boundaries. a Distance to the nearest boundary between replicates. b Venn diagrams show the overlap of TAD boundaries between replicates for control or Srrm2 knockdown cells. [file 13072_2019_289_MOESM5_ESM.jpg]

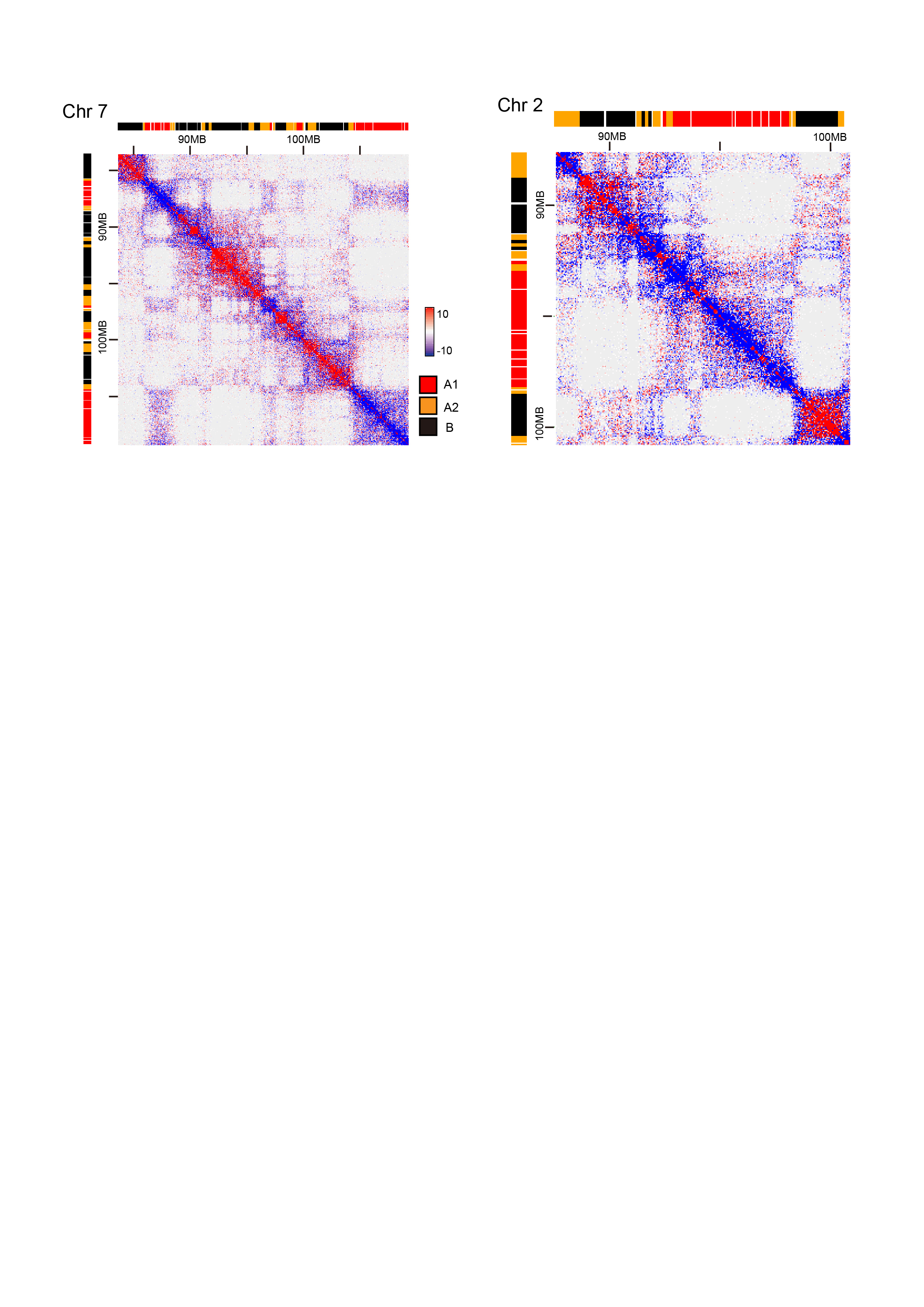

Supplement: Supplementary file 6 — Additional file 6: Figure S6. Differential contact map of intra-chromosomal inter-TADs showing fewer (compartments A, blue) and more (compartments B, red) Hi-C signal after Srrm2 depletion AML12 cells. [file 13072_2019_289_MOESM6_ESM.jpg]
